# Research and Development of a CO_2_-Responsive TMPDA–SDS–SiO_2_ Gel System for Profile Control and Enhanced Oil Recovery

**DOI:** 10.3390/gels11090709

**Published:** 2025-09-03

**Authors:** Guojun Li, Meilong Fu, Jun Chen, Yuhao Zhu

**Affiliations:** 1 College of Petroleum Engineering, Yangtze University, Wuhan 430100, China; 202071283@yangtzeu.edu.cn (G.L.); 2024710386@yangtzeu.edu.cn (Y.Z.); 2 Oil and Gas Technology Research Institute, PetroChina Qinghai Oilfield Company, Jiuquan 736202, China; chenjunqh@petrochina.com.cn

**Keywords:** CO_2_-responsive gel, heterogeneous reservoir, profile control and oil recovery enhancement

## Abstract

A CO_2_-responsive TMPDA–SDS–SiO_2_ gel system was developed and evaluated through formulation optimization, structural characterization, rheological testing, and core flooding experiments. The optimal formulation was identified as 7.39 wt% SDS, 1.69 wt% TMPDA, and 0.1 wt% SiO_2_, achieving post-CO_2_ viscosities above 10^3^–10^4^ mPa·s. Spectroscopic and microscopic analyses confirmed that CO_2_ protonates TMPDA amine groups to form carbamate/bicarbonate species, which drive the micellar transformation into a wormlike network, thereby enhancing gelation and viscosity. Rheological tests showed severe shear-thinning behavior, excellent shear recovery, and reversible viscosity changes under alternating CO_2_/N_2_ injection. The gel demonstrated rapid responsiveness, reaching stable viscosities within 8 min, and maintained good performance after 60 days of thermal aging at 90 °C and in high-salinity brines. Plugging tests in sand-packed tubes revealed that a permeability reduction of 98.9% could be achieved at 0.15 PV injection. In heterogeneous parallel core flooding experiments, the gel preferentially reduced high-permeability channel conductivity, improved sweep efficiency in low-permeability zones, and increased incremental oil recovery by 14.28–34.38% depending on the permeability contrast. These findings indicate that the CO_2_-responsive TMPDA–SDS–SiO_2_ gel system offers promising potential as a novel smart blocking gel system for improving the effectiveness of CO_2_ flooding in heterogeneous reservoirs.

## 1. Introduction

With the increasing difficulty of developing unconventional oil and gas resources, enhancing oil recovery (EOR) and extending the production life of mature reservoirs have become important challenges in the petroleum industry. CO_2_ flooding has attracted wide attention due to its dual advantages of improving recovery efficiency and contributing to carbon sequestration [1,2,3]. However, because of reservoir heterogeneity and the unfavorable mobility ratio between CO_2_ and crude oil, CO_2_ tends to finger through high-permeability channels during displacement, resulting in limited sweep efficiency and low displacement efficiency. These issues have to some extent restricted the practical effectiveness of CO_2_ flooding [4,5,6,7,8].

To improve both sweep efficiency and displacement efficiency in CO_2_ flooding, various mobility-control strategies have been proposed, including water-alternating-gas (WAG) injection [9,10,11,12,13], CO_2_ foam flooding [14,15], polymer-assisted CO_2_ flooding [16], and CO_2_ thickening techniques [17,18]. On this basis, several modified approaches have been developed in recent years. For example, chemically enhanced WAG introduces chemical agents into the water slug, which interact with subsequently injected CO_2_ through chemical reactions or interfacial stabilization, thereby improving fluid transport characteristics [19]. Another example is nanocomposite viscosity-enhancing systems, in which nanoparticles and polymers act synergistically to increase solution viscosity, delay CO_2_ breakthrough in high-permeability channels, and demonstrate potential applicability in WAG or gas flooding processes [20]. These methods not only alleviate CO_2_ channeling and improve sweep efficiency but may also enhance displacement efficiency by reducing interfacial tension or increasing relative pressure gradients, thereby contributing to further improvements in oil recovery.

Among these methods, gel systems have been reported to show potential for mitigating CO_2_ channeling in high-permeability zones due to their plugging capability and adaptability to reservoir temperature and pressure conditions. In recent years, CO_2_-responsive gels, which undergo sol–gel transitions upon CO_2_ stimulation, have been regarded as promising smart fluids for improving the effectiveness of CO_2_ flooding [21]. Such gels can undergo structural transformations under CO_2_ exposure, enabling relatively selective plugging and flow redirection. In contrast, traditional gels or nanocomposite viscosity-enhancing systems mainly rely on high pre-injection viscosity to improve the mobility ratio, which may limit injectivity and selectivity. CO_2_-responsive gels, however, can be injected as low-viscosity solutions and subsequently undergo in situ gelation under CO_2_ stimulation, making them more suitable for blocking high-permeability channels.

Previous studies have reported promising results for CO_2_-responsive gels under different conditions. For instance, a carrageenan–PEI–ethylenediamine system was reported to form a three-dimensional network structure under CO_2_ exposure, exhibiting strong plugging capability and possible oil recovery enhancement [22]. CO_2_-responsive preformed particle gels demonstrated strong swelling capacity and plugging strength under acidic environments, achieving nearly complete plugging in core flooding experiments [23]. Furthermore, a CO_2_-responsive gel–wormlike micelle coupling system exhibited significant viscosity enhancement and improved displacement performance upon CO_2_ stimulation [24]. In addition, PEI–SDS gels containing nano-SiO_2_ were reported to display favorable rheological reinforcement and plugging ability [25]. More recently, Xin and co-workers investigated the application of CO_2_-responsive gels in fractured and low-permeability reservoirs, suggesting their potential applicability under complex heterogeneous conditions [26].

In this context, a CO_2_-responsive TMPDA–SDS–SiO_2_ gel system was developed in this study. The formulation, rheological properties, and CO_2_-triggered behavior of the gel were characterized, and parallel core flooding experiments were conducted under permeability contrasts of 10, 20, and 30 to examine its plugging and displacement features under heterogeneous conditions. Building on previous work, this study provides additional experimental observations regarding the applicability of the CO_2_-responsive TMPDA–SDS–SiO_2_ gel system under heterogeneous conditions.

## 2. Results and Discussion

### 2.1. Formulation Optimization

#### 2.1.1. Effect of SDS to TMPDA Molar Ratio on System Viscosity

**Figure 1:**
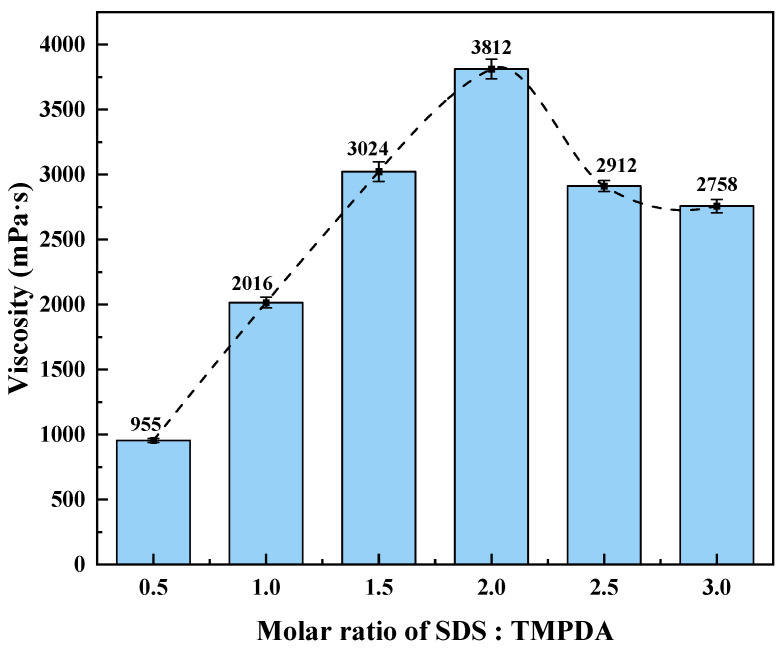
Viscosity of the CO_2_-responsive TMPDA–SDS–SiO_2_ gel system at different SDS:TMPDA molar ratios after CO_2_ introduction. Detailed data are provided in Table A1.

At a fixed total molar concentration of 300 mmol/L, the viscosity response of the CO_2_-responsive TMPDA–SDS–SiO_2_ gel system was evaluated at SDS:TMPDA molar ratios of 0.5, 1.0, 1.5, 2.0, 2.5, and 3.0. Before CO_2_ introduction, the solution exhibited a viscosity of approximately 1.2 mPa·s. After CO_2_ stimulation, the viscosities increased to 955, 2016, 3024, 3812, 2912, and 2758 mPa·s, respectively, showing an overall trend of first increasing and then decreasing (Figure 1). The maximum viscosity (3812 mPa·s) was obtained at a molar ratio of 2:1, suggesting that this ratio provided the most favorable conditions for viscosity enhancement.

This peak viscosity can be explained by the micellar structural transformation induced by compositional balance. At the 2:1 ratio, TMPDA acts as a divalent organic cation that promotes the growth of elongated wormlike micelles with greater flexibility, thereby enhancing the probability of entanglement into a transient three-dimensional network and significantly increasing viscosity [27,28]. Similar micelle growth and viscosity enhancement phenomena have been reported in other surfactant–amine systems [29,30]. When the SDS fraction was further increased, excess anionic surfactant tended to form spherical or short-rod micelles, which reduced micelle entanglement and led to a decline in viscosity [31].

#### 2.1.2. Effect of SDS–TMPDA Mass Fraction on System Viscosity

**Figure 2:**
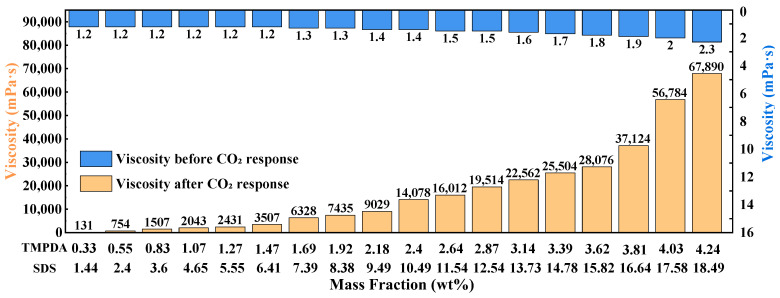
Viscosity variation in the CO_2_-responsive TMPDA–SDS–SiO_2_ gel system before and after CO_2_ introduction at different mass fractions. Detailed data are provided in Table A2.

Based on the optimal SDS:TMPDA molar ratio of 2:1 determined in Section 2.1.1, the influence of total mass fraction (1.44 wt% SDS + 0.33 wt% TMPDA to 18.49 wt% SDS + 4.24 wt% TMPDA) on viscosity was further investigated (Figure 2). Before CO_2_ introduction, viscosities remained low and nearly constant (1.2–2.3 mPa·s). After CO_2_ stimulation, however, viscosities increased markedly, from 131 mPa·s at the lowest concentration to 67,890 mPa·s at the highest concentration.

This sharp increase can be attributed to TMPDA protonation, which enhances intermolecular interactions and promotes cross-linked micellar network formation. Higher concentrations provide more micelles and greater entanglement density, further reinforcing the gel structure. Nevertheless, excessively high concentrations, despite producing higher viscosities, would increase material cost and injection pressure, which may hinder field application. Balancing performance and feasibility, the composition of 7.39 wt% SDS + 1.69 wt% TMPDA was selected for subsequent experiments.

#### 2.1.3. Effect of Nano-Silica on the Viscosity of the System

**Figure 3:**
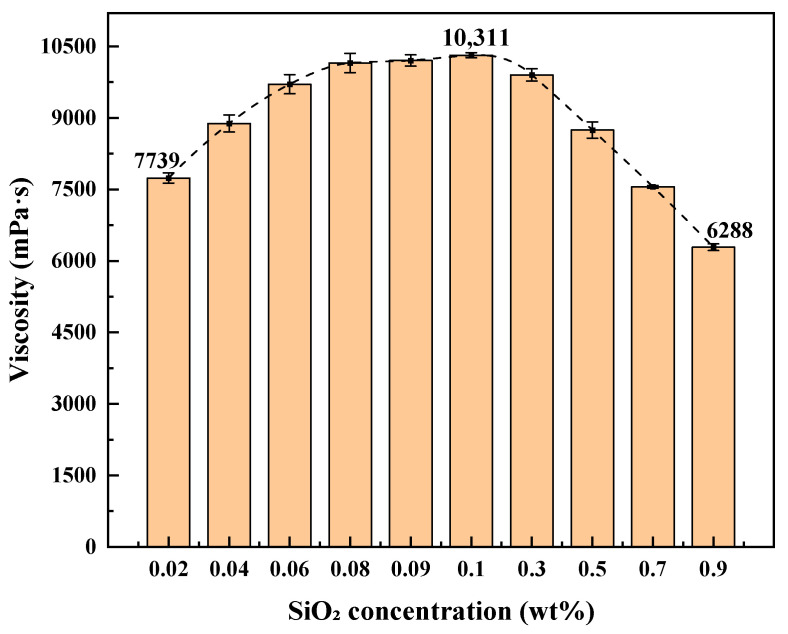
Viscosity of TMPDA–SDS–SiO_2_ systems at varying nano-silica mass fractions. Detailed experimental data are provided in Table A3.

Based on the optimized formulation of 7.39 wt% SDS and 1.69 wt% TMPDA, the effect of nano-silica concentration on viscosity was investigated (Figure 3). As the nano-silica content increased from 0.02 wt% to 0.1 wt%, the viscosity rose from 7739 mPa·s to a maximum of 10,311 mPa·s. When the concentration exceeded 0.1 wt%, viscosity decreased continuously, reaching 6288 mPa·s at 0.9 wt%.

These results suggest a concentration-dependent dual effect of nano-silica. At low dosages (<0.1 wt%), well-dispersed nanoparticles act as crosslinking points, strengthening the micellar network through hydrogen bonding, electrostatic attraction, and interfacial interactions, thus enhancing viscosity. At higher dosages (>0.1 wt%), nanoparticle aggregation and competitive adsorption disturb network homogeneity and weaken micellar entanglement, leading to reduced viscosity. Taking both viscosity enhancement and stability into account, 0.1 wt% was identified as the optimal nano-silica concentration. Therefore, the final optimized formulation of the CO_2_-responsive TMPDA–SDS–SiO_2_ gel system was 7.39 wt% SDS, 1.69 wt% TMPDA, and 0.1 wt% SiO_2_.

### 2.2. Xiangyingjili Fenxi

#### 2.2.1. Spectroscopic Evidence of CO_2_-Induced Interactions (^1^H-NMR and FTIR)

**Figure 4:**
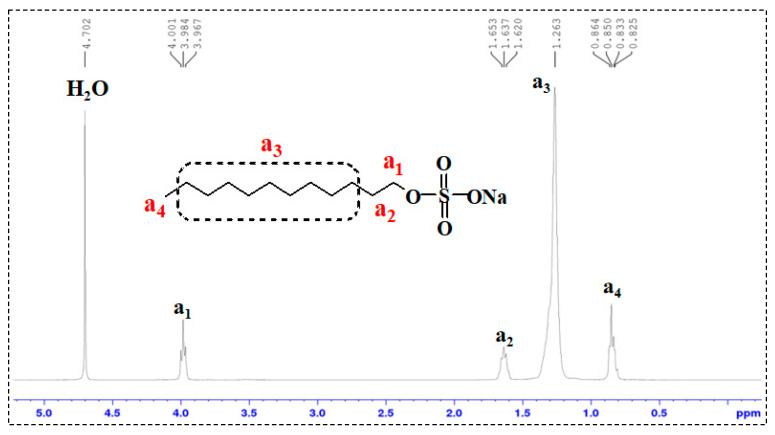
^1^H-NMR spectrum of SDS.

**Figure 5:**
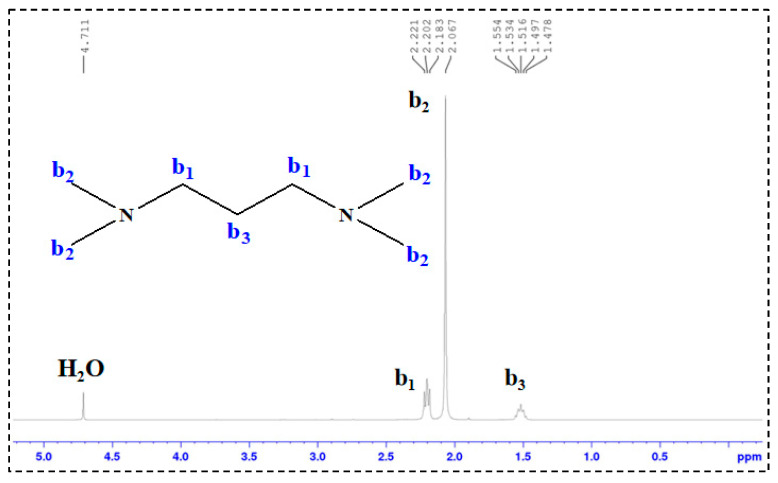
^1^H-NMR spectrum of TMPDA.

**Figure 6:**
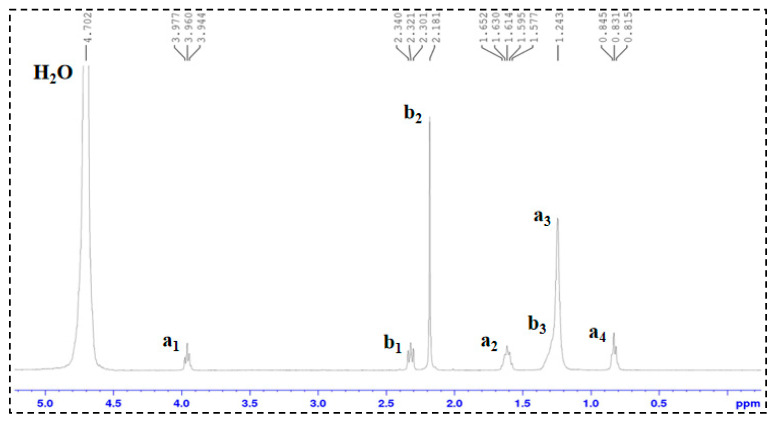
^1^H-NMR spectrum of the TMPDA–SDS binary system.

**Figure 7:**
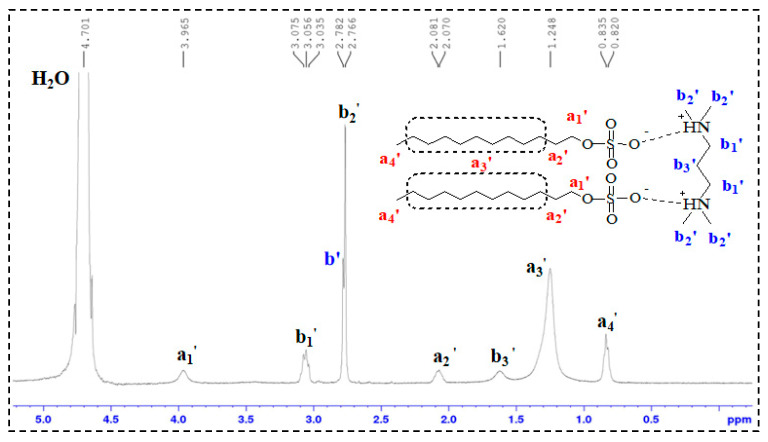
^1^H-NMR spectrum of the CO_2_-responsive TMPDA–SDS system after CO_2_ stimulation.

**Figure 8:**
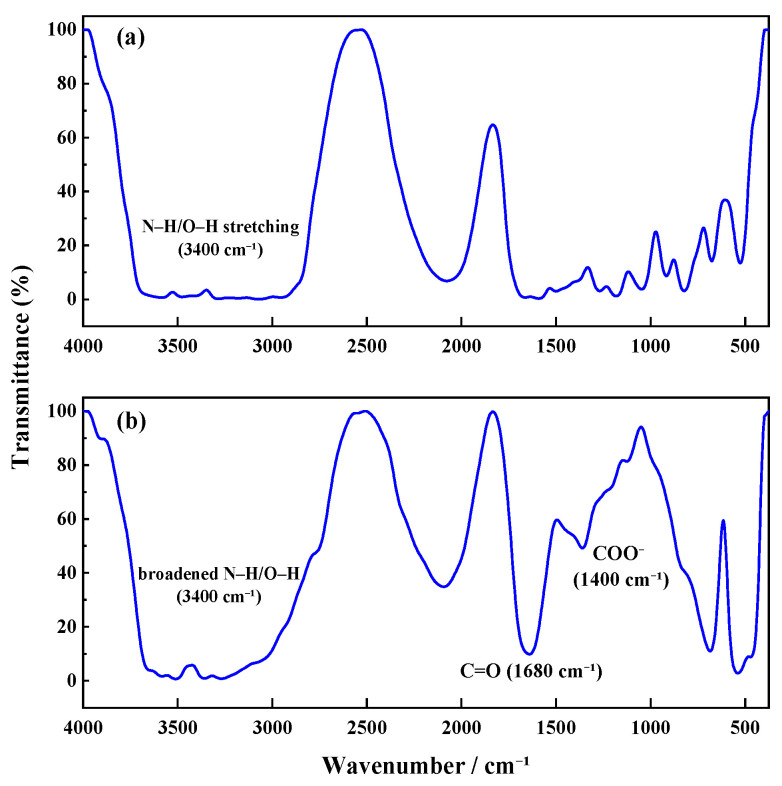
FTIR spectra of the TMPDA–SDS system before (**a**) and after (**b**) CO_2_ introduction.

To elucidate the CO_2_-triggered structural changes in the TMPDA–SDS system, both ^1^H nuclear magnetic resonance (^1^H-NMR) and Fourier transform infrared (FTIR) spectroscopy were employed (Figure 4, Figure 5, Figure 6, Figure 7 and Figure 8).

In the ^1^H-NMR spectra of SDS and TMPDA (Figure 4 and Figure 5), characteristic resonances corresponding to alkyl chains and amine groups were clearly observed. Upon mixing SDS and TMPDA (Figure 6), no significant shifts or new peaks appeared, suggesting that their interactions are dominated by non-covalent forces such as electrostatic attraction and hydrophobic association rather than covalent bonding. However, after CO_2_ introduction (Figure 7), distinct spectral changes emerged. Specifically, new peaks (a_1_′, b_1_′) appeared adjacent to the original SDS and TMPDA signals, accompanied by downfield shifts and broadening of existing peaks. These spectral modifications indicate the generation of new proton environments, consistent with the reaction of CO_2_ with the amine groups of TMPDA. Such changes suggest the possible formation of CO_2_-derived carbamate or bicarbonate species, although ^1^H-NMR alone cannot unambiguously distinguish between them.

The FTIR spectra provide complementary and more direct evidence. Before CO_2_ introduction (Figure 8), the TMPDA–SDS system exhibited no distinctive absorption in the carbonyl region. After CO_2_ exposure, two new strong bands appeared near ~1680 cm^−1^ and ~1400 cm^−1^, which can be assigned to C=O stretching and COO^−^ symmetric/asymmetric vibrations, respectively. These features are characteristic of carbamate and bicarbonate species, thereby confirming that CO_2_ reacts with TMPDA to generate such ionic derivatives.

Taken together, the combined NMR and FTIR results demonstrate that CO_2_ protonates the TMPDA amine groups and leads to the formation of carbamate and/or bicarbonate species. These ionic products alter the charge balance and molecular interactions in the TMPDA–SDS–CO_2_ system, promoting micellar restructuring and the transition toward entangled wormlike networks that underpin the macroscopic gelation and viscosity enhancement.

#### 2.2.2. Morphological Evolution from SEM Observations

**Figure 9:**
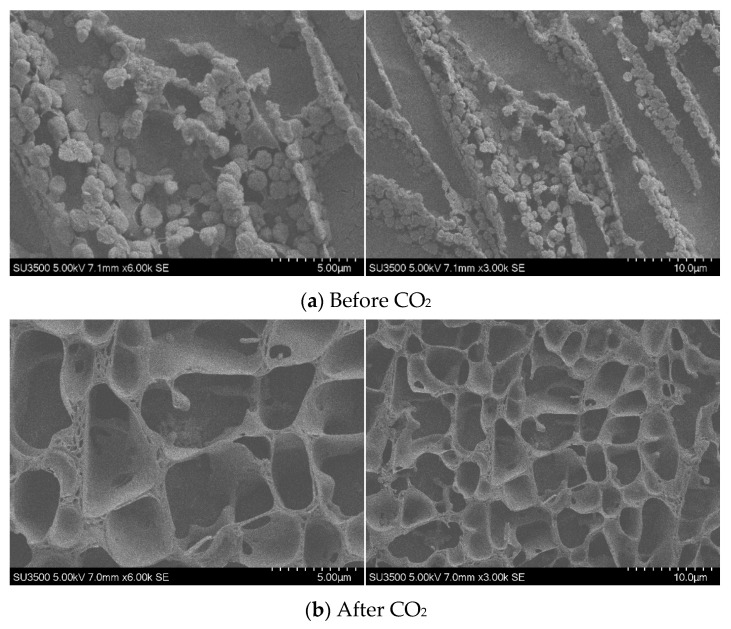
SEM images of the TMPDA–SDS system before (**a**) and after (**b**) CO_2_ introduction.

Cryo-SEM was employed to observe the morphological changes in the TMPDA–SDS system before and after CO_2_ stimulation (Figure 9).

Before CO_2_ introduction (Figure 9a), the samples exhibited a loose and discontinuous morphology, consisting of irregular aggregates with poorly connected structures. Such features indicate insufficient micelle entanglement and limited network formation, consistent with the low viscosity measured in rheological tests. After CO_2_ introduction (Figure 9b), the micrographs revealed a significant transformation into a denser, sponge-like network with highly interconnected pores. The emergence of this continuous three-dimensional framework demonstrates enhanced intermolecular associations and structural reorganization under CO_2_ stimulation, which accounts for the substantial viscosity increase in the CO_2_-responsive TMPDA–SDS system.

### 2.3. Rheological Behavior of the CO_2_-Responsive Gel

#### 2.3.1. Rheological Properties Before and After CO_2_ Response

**Figure 10:**
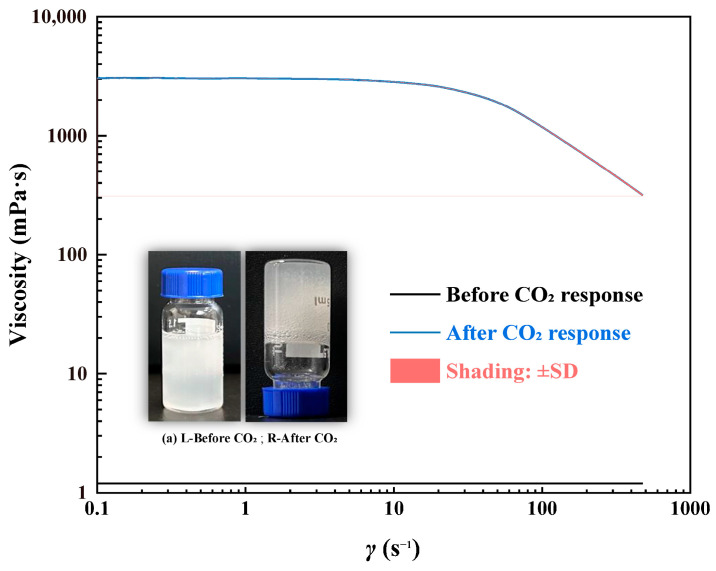
Rheological properties of the CO_2_-responsive TMPDA–SDS–SiO_2_ gel system before and after CO_2_ exposure. Photographs showing the sol–gel transition. Detailed experimental data are provided in Table A4.

As shown in Figure 10, the TMPDA–SDS–SiO_2_ gel system exhibits distinctly different rheological behavior before and after CO_2_ exposure. Prior to CO_2_ introduction, the viscosity remained close to 1 mPa·s across the entire shear rate range, consistent with the characteristics of a Newtonian fluid. After CO_2_ stimulation, however, the viscosity rapidly increased to the order of 10^3^–10^4^ mPa·s and displayed clear shear-thinning behavior. This transition reflects the formation of a structured three-dimensional network, which resists flow at low shear rates but gradually breaks down under higher shear. The accompanying photographs (Figure 10) further illustrate this macroscopic change, showing the transformation from a transparent solution to a semi-translucent, highly viscous gel, thereby confirming the CO_2_-induced microstructural reconstruction.

#### 2.3.2. Shear–Rest–Shear Cyclic Tests

**Figure 11:**
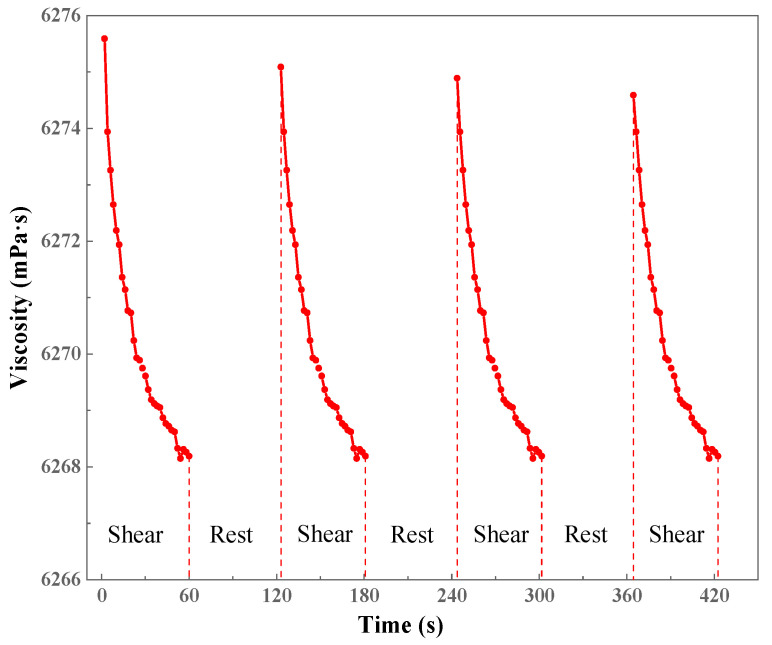
Shear–rest–shear cyclic test of the CO_2_-responsive TMPDA–SDS–SiO_2_ gel system after CO_2_ exposure.

To assess the structural reversibility of the gel network, shear–rest–shear cyclic experiments were performed on the CO_2_-responsive TMPDA–SDS–SiO_2_ gel system (Figure 11). Under high shear, the viscosity decreased sharply due to partial disruption of the micellar entanglement. Once shearing ceased, the viscosity gradually recovered, indicating that the disrupted network could spontaneously reorganize through noncovalent interactions such as electrostatic attraction, hydrogen bonding, and hydrophobic association. After several shear–rest cycles, the system largely regained its initial viscosity, demonstrating excellent shear-recovery performance. This reversibility is advantageous for field applications, where gels must endure dynamic flow conditions while maintaining plugging ability.

#### 2.3.3. Alternating CO_2_/N_2_ Injection

**Figure 12:**
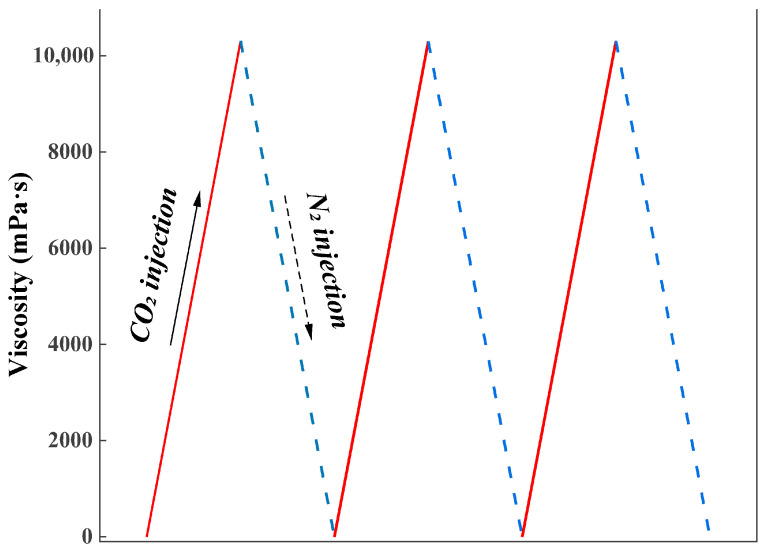
Viscosity response of the CO_2_-responsive TMPDA–SDS–SiO_2_ gel system under alternating CO_2_/N_2_ injection.

The cyclic viscosity response under alternating CO_2_/N_2_ injection is presented in Figure 12. Upon CO_2_ injection, the viscosity increased sharply, whereas subsequent N_2_ injection caused a rapid decrease, reflecting partial disassembly of the CO_2_-stabilized micellar network. The repeatable rise-and-fall profiles over multiple cycles confirm that the CO_2_-responsive TMPDA–SDS–SiO_2_ gel system exhibits reversible gas responsiveness. Mechanistically, CO_2_ protonates the amino groups of TMPDA, generating carbamate/bicarbonate ionic species that promote crosslinking with SDS micelles, thereby forming a dense wormlike network. Displacement of CO_2_ by N_2_ reverses this protonation process, weakening intermolecular associations and reducing viscosity. Such tunable rheological behavior highlights the potential for regulating plugging and unplugging functions in reservoir environments through alternating CO_2_/N_2_ stimulation.

### 2.4. Thermal and Salt Resistance of CO_2_-Responsive Gels

To further evaluate the stability and applicability of the optimized CO_2_-responsive TMPDA–SDS–SiO_2_ gel system, three aspects were investigated: the influence of CO_2_ injection time on gel viscosity, the thermal stability under high-temperature aging, and the salt tolerance in simulated formation brines.

#### 2.4.1. Effect of CO_2_ Injection Time on Gel Viscosity

**Figure 13:**
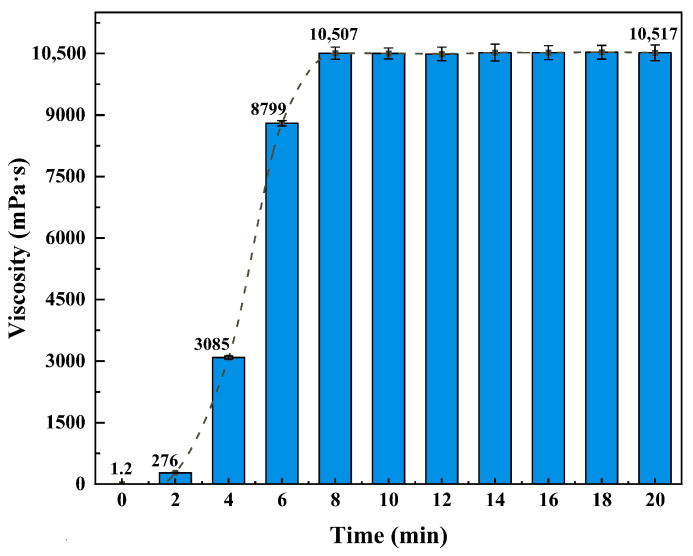
Effect of CO_2_ injection time on the viscosity evolution of the CO_2_-responsive TMPDA–SDS–SiO_2_ gel system. Detailed experimental data are provided in Table A5.

As shown in Figure 13, the viscosity of the CO_2_-responsive TMPDA–SDS–SiO_2_ gel system increased sharply with prolonged CO_2_ injection. The viscosity rose from 1.2 mPa·s at the initial stage to 10,507 mPa·s within 8 min, after which it reached a plateau and remained stable (10,517 mPa·s at 20 min). This behavior indicates that the gel achieves steady-state gelation rapidly, confirming its ability to undergo structural reconstruction within a short response time. Such fast responsiveness is advantageous for field applications where timely gelation is required under reservoir conditions.

#### 2.4.2. Thermal Aging Stability at 90 °C

**Figure 14:**
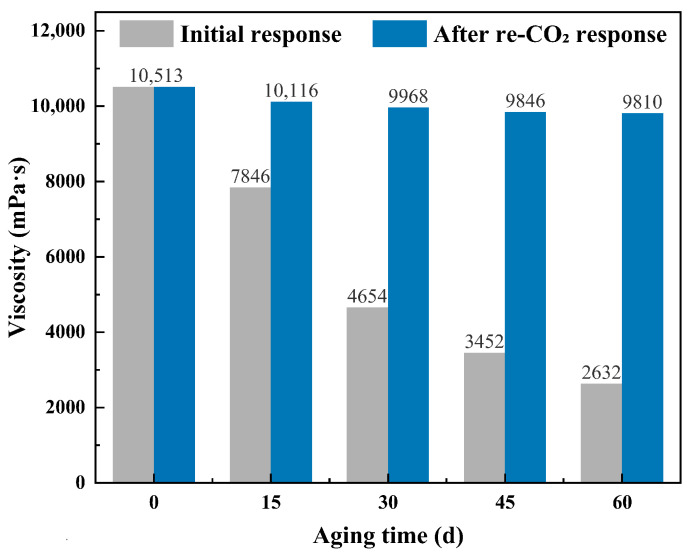
Thermal aging stability of the CO_2_-responsive TMPDA–SDS–SiO_2_ gel system at 90 °C for up to 60 days, including viscosity recovery upon re-CO_2_ exposure.

The thermal stability of the CO_2_-responsive TMPDA–SDS–SiO_2_ gel system was evaluated by aging samples at 90 °C for up to 60 days, with viscosities measured both before and after re-introduction of CO_2_ (Figure 14). The initial CO_2_-induced viscosity decreased progressively with increasing aging time, dropping from 10,513 mPa·s at day 0 to 2632 mPa·s at day 60. Nevertheless, upon re-exposure to CO_2_, the gel viscosity could be effectively recovered, maintaining values above 9800 mPa·s even after 60 days of high-temperature treatment. These results indicate that while prolonged thermal aging partially weakens the gel structure, the system exhibits excellent CO_2_-responsive recovery, ensuring long-term functional stability under reservoir conditions.

#### 2.4.3. Salt Tolerance Under Different Salinity Levels

**Figure 15:**
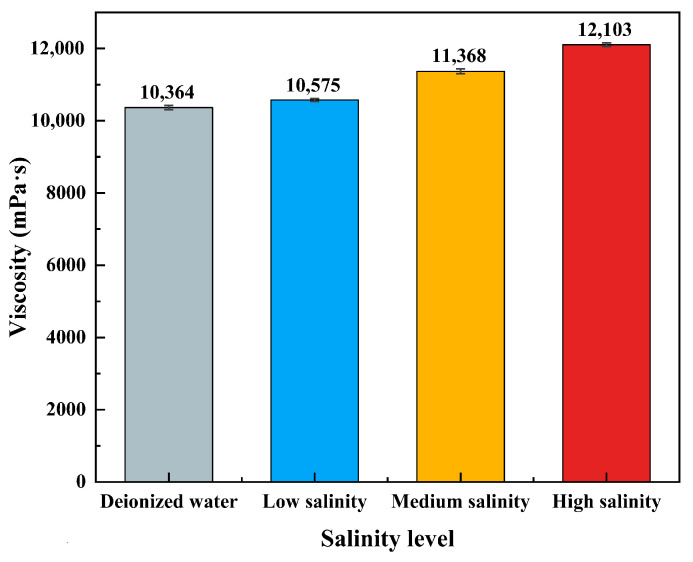
Viscosity of the CO_2_-responsive TMPDA–SDS–SiO_2_ gel system prepared in deionized water and simulated brines of different salinity levels. Detailed experimental data are provided in Table A6.

The salt tolerance of the CO_2_-responsive TMPDA–SDS–SiO_2_ gel system was evaluated using deionized water, low-salinity brine, medium-salinity brine, and high-salinity brine as solvents (Figure 15). The results showed a slight increase in viscosity with increasing salinity, from 10,364 mPa·s in deionized water to 12,103 mPa·s in high-salinity brine. This enhancement can be attributed to the screening effect of electrolytes, which reduces electrostatic repulsion between charged micelles and facilitates closer packing and reinforcement of the micellar network. These findings demonstrate that the gel system possesses excellent salt tolerance and remains effective under a wide range of reservoir salinity conditions.

### 2.5. Plugging Performance and Heterogeneous Core Flooding Tests

#### 2.5.1. Relationship Between Injected PV and Plugging Performance

The plugging performance of the CO_2_-responsive TMPDA–SDS–SiO_2_ gel system in sand-packed tubes was evaluated at different injected pore volumes (PV) under a backpressure of 5 MPa (Table 1). With increasing injected PV, permeability reduction became progressively more pronounced. The blocking efficiency rose from 74.6% at 0.1 PV to 90% at 0.14 PV and reached 98.9% when the injection volume reached 0.15 PV. These results demonstrate that the gel system can achieve nearly complete plugging at relatively low injection volumes, providing the foundation for subsequent heterogeneous core flooding experiments.

#### 2.5.2. Heterogeneous Parallel Core Flooding Experiments

**Figure 16:**
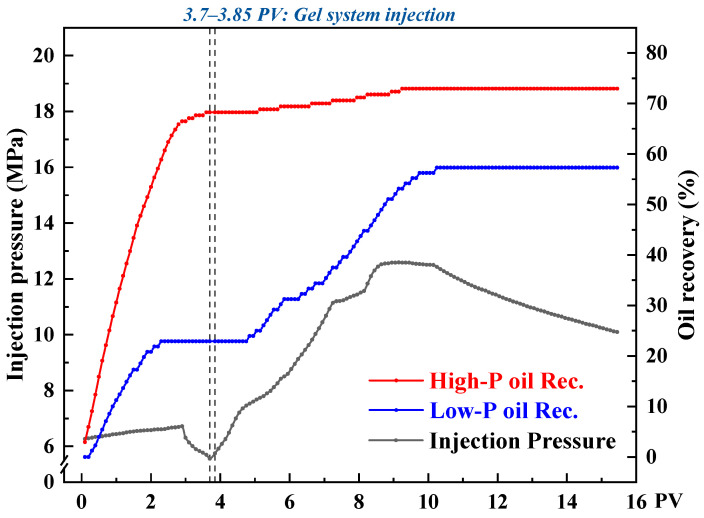
Injection pressure and recovery curves during CO_2_ flooding with the CO_2_-responsive TMPDA–SDS–SiO_2_ gel system at a permeability contrast of 10.

**Figure 17:**
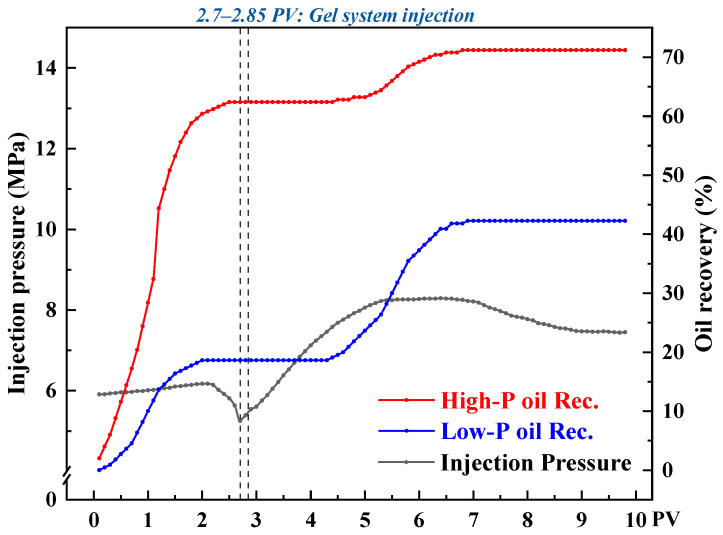
Injection pressure and recovery curves during CO_2_ flooding with the CO_2_-responsive TMPDA–SDS–SiO_2_ gel system at a permeability contrast of 20.

**Figure 18:**
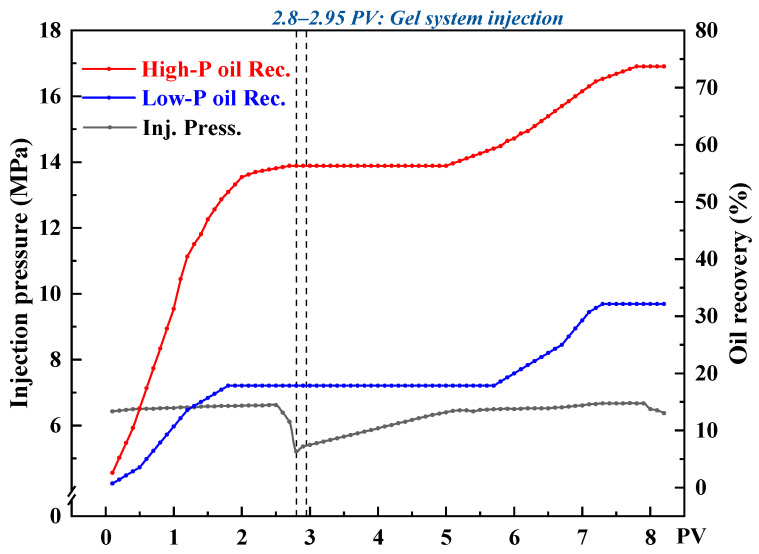
Injection pressure and recovery curves during CO_2_ flooding with the CO_2_-responsive TMPDA–SDS–SiO_2_ gel system at a permeability contrast of 30.

To investigate the flow-diverting behavior of the CO_2_-responsive TMPDA–SDS–SiO_2_ gel system under heterogeneous conditions during CO_2_ flooding, three sets of parallel core flooding experiments were conducted with permeability contrasts of 10, 20, and 30. Figure 16, Figure 17 and Figure 18 present the injection pressure and oil recovery curves, and the corresponding recovery data are summarized in Table 2.

During the initial CO_2_ flooding stage, the injected gas preferentially entered the high-permeability cores, resulting in rapid breakthrough and higher recovery factors (68.24%, 62.40%, and 56.30% for contrasts of 10, 20, and 30, respectively), while the low-permeability cores exhibited delayed breakthrough and lower recoveries (22.92%, 18.64%, and 17.86%). After injecting 0.15 PV of the CO_2_-responsive gel, the permeability of the high-permeability channels was significantly reduced, leading to a sharp rise in injection pressure. This forced subsequent CO_2_ to flow into the low-permeability regions, thereby improving their recoveries by 34.38%, 23.63%, and 14.28% under contrasts of 10, 20, and 30, respectively.

From the displacement curves, it can be seen that when the permeability contrast was low (contrast = 10), the gel was distributed more uniformly, which effectively expanded the sweep volume in the low-permeability core and yielded the highest incremental recovery. In contrast, under severe heterogeneity (contrast = 30), the dominant flow in the high-permeability path limited gel penetration into the low-permeability zone, resulting in a lower recovery increment.

Overall, the CO_2_-responsive gel system exhibited a clear profile control effect by preferentially reducing the permeability of high-permeability channels, suppressing CO_2_ channeling, and improving oil recovery in low-permeability regions. As the degree of heterogeneity increased, the profile control efficiency declined, indicating that in field applications, the gel dosage and plugging strategies should be adjusted according to permeability distribution to improve overall recovery.

## 3. Conclusions

In this study, a CO_2_-responsive TMPDA–SDS–SiO_2_ gel system was developed and evaluated. The main findings are as follows:

(1) Formulation optimization: The optimal composition was identified as 7.39 wt% SDS + 1.69 wt% TMPDA + 0.1 wt% SiO_2_. This system exhibited rapid CO_2_-triggered gelation, with viscosity increasing from 1.2 mPa·s to >10,000 mPa·s within 8 min.
(2) Rheological performance: The gel showed reversible shear-thinning behavior, good shear recovery, and repeatable responsiveness under alternating CO_2_/N_2_ injection, suggesting adaptability under dynamic flow conditions.
(3) Stability: The gel retained high viscosity after thermal aging (>9800 mPa·s after 60 days at 90 °C) and maintained effectiveness across a wide salinity range.
(4) Plugging and flooding performance: In sand-packed tubes, plugging efficiency reached 98.9% at 0.15 PV injection. In heterogeneous dual-core flooding tests, incremental recoveries of 34.4%, 23.6%, and 14.3% were obtained at permeability contrasts of 10, 20, and 30, respectively.

In summary, the TMPDA–SDS–SiO_2_ gel system exhibited fast CO_2_ responsiveness, satisfactory long-term stability, and effective profile control in laboratory tests, indicating its potential for improving mobility control and oil recovery in heterogeneous reservoirs.

## 4. Materials and Methods

### 4.1. Experimental Materials and Equipment

Experimental Materials: The low-molecular-weight amine used in this study was N,N,N′,N′-tetramethyl-1,3-propanediamine (TMPDA, analytical grade). The surfactant was sodium dodecyl sulfate (SDS, analytical grade). The nano-silica consisted of spherical particles with an average diameter of approximately 20 nm. Carbon dioxide (CO_2_, 99 mol%) was used as the triggering gas. Analytical-grade sodium chloride (NaCl), calcium chloride (CaCl_2_), and magnesium chloride (MgCl_2_) were used to prepare synthetic brines. Deionized water was used in all experiments. The oil sample was a mixture of crude oil from the Zhongyuan Oilfield and kerosene at a 1:1 mass ratio, with the viscosity of the simulated oil measured as 3.08 mPa·s at 40 °C.

**Figure 19:**
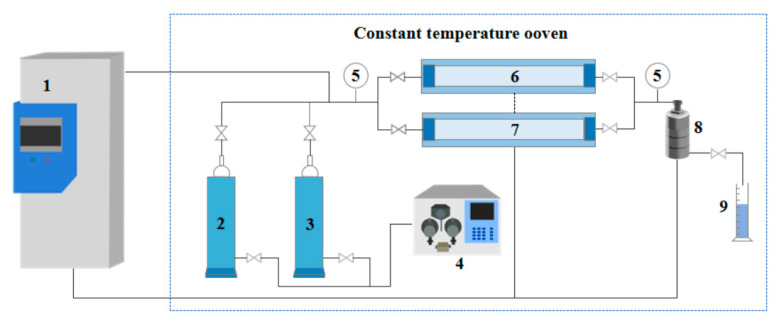
Core flooding experiment setup. (1) CO_2_ injection pump; (2) simulated oil/water; (3) CO_2_-responsive TMPDA–SDS–SiO_2_ solution; (4) constant flow and pressure displacement pump; (5) pressure gauge; (6–7) high- and low-permeability core holders; (8) backpressure valve; (9) gas–liquid separator.

Experimental Equipment: The main instruments included a digital viscometer (DV-II, Brookfield, AMETEK, Middleboro, MA, USA), a flowmeter (G10-15F, Chengfeng Flowmeter Co., Ltd., Shandong, China), a nuclear magnetic resonance (NMR) spectrometer (Avance III 300 MHz, Bruker Corporation, Billerica, MA, USA), a rotational rheometer (Haake MARS 60, Thermo Fisher Scientific, Karlsruhe, Germany), a scanning electron microscope (SU3500, Hitachi High-Tech Corporation, Tokyo, Japan), and a Fourier transform infrared (FTIR) spectrometer (Tensor 27, Bruker Corporation, Billerica, MA, USA). A core flooding system (see Figure 19) was employed to conduct heterogeneous dual-core parallel flooding experiments.

### 4.2. Preparation and Optimization of the CO_2_-Responsive TMPDA–SDS–SiO_2_ Gel System

Gel Preparation Method: SDS, TMPDA, and nano-silica were dissolved in deionized water in the designed proportions and placed in a three-necked flask. The mixture was stirred at room temperature for 5 min until a transparent solution was obtained, representing the pre-gelation state. After standing for 10 min, the solution was transferred to a gas-washing bottle. Under ambient temperature and pressure, CO_2_ was continuously introduced at a flow rate of 0.1 L·min^−1^ using a glass rotameter, while stirring was maintained to ensure adequate gas–liquid contact until the solution transformed into a viscoelastic gel.

Formulation Optimization Method: A single-factor method was used to evaluate the effects of the SDS:TMPDA molar ratio, the total mass fraction of SDS + TMPDA, and the nano-silica content on the viscosity of the CO_2_-responsive TMPDA–SDS–SiO_2_ gel system, with the goal of determining the optimal formulation. The experimental design included the following: (1) SDS:TMPDA molar ratios of 0.5, 1.0, 1.5, 2.0, 2.5, and 6.0; (2) 18 groups of total SDS + TMPDA mass fractions, ranging from 1.44% SDS + 0.33% TMPDA to 18.49% SDS + 4.24% TMPDA; and (3) nano-silica concentrations of 0.02%, 0.04%, 0.06%, 0.08%, 0.09%, 0.1%, 0.3%, 0.5%, 0.7%, and 0.9%. The viscosity of the gel after CO_2_ stimulation was measured using a digital viscometer.

### 4.3. Structural Characterization

^1^H-NMR Analysis: ^1^H nuclear magnetic resonance (^1^H-NMR) spectroscopy was performed on TMPDA, SDS, a TMPDA–SDS mixture, and the CO_2_-responsive TMPDA–SDS mixture. The procedure was as follows: (1) each sample was dissolved in D_2_O and transferred to an NMR tube, with the measurement temperature set at 25 °C; (2) the NMR probe was tuned to match the magnetic field and radio frequency source; (3) the instrument was calibrated and parameters were set; and (4) data were acquired, and free induction decay (FID) signals were collected.

FTIR Analysis: Fourier-transform infrared (FTIR) spectroscopy was used to characterize TMPDA–SDS solutions before and after CO_2_ stimulation. Samples were tested using the smear method, and spectra were recorded at 25 °C.

SEM Analysis: Scanning electron microscopy (SEM) was employed to observe TMPDA–SDS mixtures before and after CO_2_ stimulation. The procedure included the following: (1) ultra-low-temperature freezing of the samples; (2) fracturing of the frozen samples under cryogenic conditions, followed by sputter-coating with a thin metal layer; and (3) low-temperature SEM imaging.

All structural and spectroscopic characterizations in this section were conducted on the TMPDA–SDS system with a composition of 7.39 wt% SDS and 1.69 wt% TMPDA, without the addition of nano-silica.

### 4.4. Rheological Behavior Evaluation

Steady Shear Behavior: A gel sample prepared with the optimized TMPDA–SDS–SiO_2_ formulation (7.39 wt% SDS, 1.69 wt% TMPDA, and 0.1 wt% SiO_2_) was tested for viscosity under varying shear rates (0.1–500 s^−1^) at 25 °C using a rotational rheometer.

Shear Recovery Property: The shear recovery performance was evaluated by conducting a shear–rest–shear cycle test at a constant shear rate of 100 s^−1^.

Alternating CO_2_/N_2_ Injection Test: To examine gas responsiveness, CO_2_ and N_2_ were alternately injected into the gel solution at a flow rate of 0.1 L·min^−1^ at 25 °C. Viscosity changes during gas alternation were monitored using a digital viscometer.

Effect of CO_2_ Injection Time: The influence of CO_2_ injection duration on system viscosity was investigated at 25 °C using a digital viscometer. CO_2_ was continuously injected at 0.1 L·min^−1^ for up to 20 min, and viscosity was recorded at 2 min intervals.

### 4.5. Thermal and Salt Resistance

Thermal Aging Test (90 °C): Experiments were performed on the CO_2_-responsive TMPDA–SDS–SiO_2_ gel system with the optimized formulation (7.39 wt% SDS, 1.69 wt% TMPDA, and 0.1 wt% SiO_2_). Gel samples were sealed and aged in an oven at 90 °C for 0, 15, 30, 45, and 60 days. At each aging time point, the samples were cooled to 25 °C, and their apparent viscosities were measured under identical conditions using a digital viscometer. Immediately afterward, CO_2_ was introduced into the same samples at 25 °C at a rate of 0.1 L·min^−1^ until a stable viscosity was obtained, and the post-response viscosity was recorded.

Salt Tolerance: Synthetic brines with low (5275.1 mg·L^−1^), medium (21,000 mg·L^−1^), and high (52,780.1 mg·L^−1^) salinities were prepared using NaCl, CaCl_2_, and MgCl_2_, with deionized water as the zero-salinity control. Gel solutions were formulated using these brines, and CO_2_ was injected at 25 °C at a rate of 0.1 L·min^−1^ until the viscosity stabilized. The apparent viscosity was then measured at 25 °C using a digital viscometer.

### 4.6. Plugging Performance and Heterogeneous Core Flooding

Relationship between Injected PV and Plugging Efficiency: Experiments were conducted in sand-packed tubes at 40 °C under a backpressure of 5 MPa and a confining pressure of 20 MPa, using the CO_2_-responsive TMPDA–SDS–SiO_2_ gel system (7.39 wt% SDS, 1.69 wt% TMPDA, and 0.1 wt% SiO_2_). The procedure was as follows: (1) Quartz sand was packed into the tubes, and the pore volume (PV) was determined by water saturation; (2) Initial CO_2_ permeability (k_0_) was measured at an injection rate of 2 mL·min^−1^; (3) Gel solutions were injected at different pore volumes (0.10, 0.12, 0.14, 0.15, 0.25, and 0.50 PV); (4) CO_2_ was reinjected at 2 mL·min^−1^ while continuously monitoring inlet/outlet pressures and outlet flow rate. Once steady state was reached, permeability after gel injection (k_1_) was calculated. Plugging efficiency was then determined using the plugging ratio (1–k_1_/k_0_).

Heterogeneous Dual-Core Parallel Flooding: These experiments were performed in dual-core systems with permeability contrast ratios of 10 (high: 300 mD; low: 30 mD), 20 (high: 600 mD; low: 30 mD), and 30 (high: 600 mD; low: 20 mD). The procedure was as follows: (1) High- and low-permeability cores were oven-dried and aged for 6 h, then sequentially saturated with deionized water and simulated oil; (2) The cores were mounted in the flooding apparatus, with the experimental conditions set to 40 °C, 5 MPa backpressure, and 20 MPa confining pressure; (3) CO_2_ was injected at 2 mL·min^−1^ until no further fluid was produced; (4) 0.15 PV of the TMPDA–SDS–SiO_2_ solution was injected; (5) A second CO_2_ flooding was carried out until no additional fluid was produced, concluding the experiment. Throughout the entire process, inlet/outlet pressures, cumulative oil production, and CO_2_ volume were recorded at every 0.1 PV of injection.

**Figure 20:**
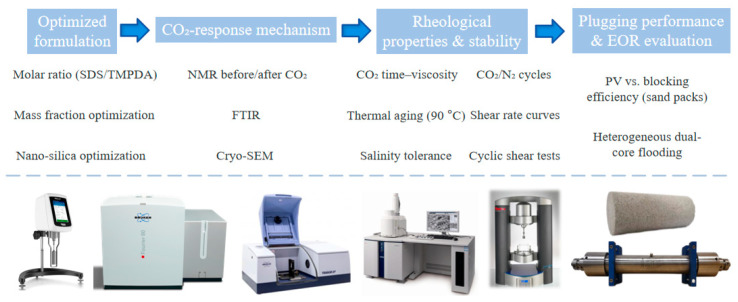
Experimental workflow of the CO_2_-responsive TMPDA–SDS–SiO_2_ gel study.

A schematic flowchart of the experimental procedures, including gel preparation, structural and rheological characterization, and core-flooding tests, is shown in Figure 20.

## Figures and Tables

**Table 1:** Plugging efficiency of the CO_2_-responsive TMPDA–SDS–SiO_2_ gel system at different injected PV values in sand-packed tubes.

PV	Porosity (%)	Initial Permeability (mD)	Permeability After Gel Injection (mD)	Blocking Efficiency (%)
0.1	41.58	435	110.4	74.6
0.12	40.27	421	72.11	82.9
0.14	40.53	428	42.67	90
0.15	42.13	448	4.74	98.9
0.25	42.01	442	3.71	99.1
0.5	41.28	425	2.42	99.4

**Table 2:** Oil recovery under different permeability contrasts before and after injection of the CO_2_-responsive TMPDA–SDS–SiO_2_ gel system.

Permeability Contrast	First CO_2_ Flooding Recovery (%)		Second CO_2_ Flooding Recovery (%)		Incremental Recovery (%)	
	High Perm.	Low Perm.	High Perm.	Low Perm.	High Perm.	Low Perm.
10	68.24	22.92	72.94	57.29	4.71	34.38
20	62.40	18.64	71.20	42.27	8.80	23.63
30	56.30	17.86	73.69	32.14	17.39	14.28

## Data Availability

The data supporting the findings of this study are available within the article. Further inquiries can be directed to the corresponding author.

## Nomenclature

**Abbreviation**	
TMPDA	N,N,N′,N′-tetramethyl-1,3-propanediamine
SDS	Sodium dodecyl sulfate
SiO_2_	Nano-silica
CO_2_	Carbon dioxide
N_2_	Nitrogen
NMR	Nuclear magnetic resonance
FTIR	Fourier transform infrared spectroscopy
SEM	Scanning electron microscopy
**Symbols**	
PV	pore volume
k_0_	initial permeability (mD)
k_1_	permeability after gel treatment (mD)
wt%	weight percent
mol%	mole percent

## Appendix A

**Table A1:** Experimental data for Figure 3.

SDS:TMPDA Molar Ratios	Viscosity (mPa·s)		
	Experiment #1	Experiment #2	Experiment #3
0.5:1	953	971	940
1:1	2011	1978	2060
1.5:1	3023	3101	2950
2:1	3807	3740	3889
2.5:1	2912	2870	2955
3:1	2754	2709	2811

**Table A2:** Experimental data for Figure 4.

SDS and TMPDA Mass Fractions	Experiment #1		Experiment #2		Experiment #3	
	Viscosity Before Response (mPa·s)	Viscosity After Response (mPa·s)	Viscosity Before Response (mPa·s)	Viscosity After Response (mPa·s)	Viscosity Before Response (mPa·s)	Viscosity After Response (mPa·s)
1.44% SDS + 0.33% TMPDA	1.2	131	-	-	-	-
2.4% SDS + 0.55% TMPDA	1.2	54	-	-	-	-
3.6% SDS + 0.83% TMPDA	1.2	1507	-	-	-	-
4.65% SDS + 1.07% TMPDA	1.2	2043	-	-	-	-
5.55% SDS + 1.27% TMPDA	1.2	2432	1.2	2449	1.2	2413
6.41% SDS + 1.47% TMPDA	1.2	3514	1.2	3509	1.2	3498
7.39% SDS + 1.69% TMPDA	1.3	6345	1.3	6317	1.3	6321
8.38% SDS + 1.92% TMPDA	1.3	7453	1.3	7432	1.3	7419
9.49% SDS + 2.18% TMPDA	1.4	9026	1.4	9043	1.4	9017
10.49% SDS + 2.4% TMPDA	1.4	14,078	-	-	-	-
11.54% SDS + 2.64% TMPDA	1.5	16,012	-	-	-	-
12.54% SDS + 2.87% TMPDA	1.5	19,514	-	-	-	-
13.73% SDS + 3.14% TMPDA	1.6	22,562	-	-	-	-
14.78% SDS + 3.39% TMPDA	1.7	25,504	-	-	-	-
15.82% SDS + 3.62% TMPDA	1.8	28,076	-	-	-	-
16.64% SDS + 3.81% TMPDA	1.9	37,124	-	-	-	-
17.58% SDS + 4.03% TMPDA	2	56,784	-	-	-	-
18.49% SDS + 4.24% TMPDA	2.3	67,890	-	-	-	-

**Table A3:** Experimental data for Figure 5.

SiO_2_ Mass Fraction (%)	Viscosity (mPa·s)		
	Experiment #1	Experiment #2	Experiment #3
0.02	7772	7657	7828
0.04	8883	8805	9026
0.06	9767	9482	9870
0.08	10,152	9981	10,255
0.09	10,271	10,065	10,276
0.1	10,311	10,259	10,369
0.3	9865	9791	10,043
0.5	8610	8690	8936
0.7	7524	7554	7583
0.9	6300	6213	6352

**Table A4:** Experimental data for Figure 12.

Shear Rate (s^−1^)	Viscosity (mPa·s)		
	Experiment #1	Experiment #2	Experiment #3
0.08282	2941.92576	2924.45198	2910.293494
-	2941.92576	2921.55696	2907.085341
-	2909.99723	2885.954902	2933.387979
-	2913.55515	2930.455156	2917.115085
-	2926.46599	2949.25395	2901.087358
-	2939.5	2948.72901	2932.001891
-	2943.61615	2951.002683	2930.950206
-	2945.05692	2949.248587	2935.054047
-	2951.07532	2952.845325	2984.947004
-	2955.93786	2954.661374	2958.943709
-	2959.50174	2971.070258	2969.89688
-	2961.24673	2960.811073	2961.884063
-	2964.08415	2968.338134	2970.802287
-	2967.90719	2969.874542	2966.743545
-	2971.13371	2976.202554	2962.736465
-	2971.97056	2979.475188	2986.780223
-	2973.99378	2975.507067	2978.762815
-	2976.16482	2974.221103	2973.830323
-	2978.1	2979.585742	2966.59278
-	2979.23088	2982.64261	2980.407619
-	2980.34668	2979.57608	2978.808597
-	2982.81312	2981.489619	2981.562697
-	2985.2	2985.835865	2981.425908
-	2986.19723	2989.655046	2976.365261
-	2987.69546	2987.156467	2988.338924
-	2989.48097	2990.49282	2989.415143
-	2991.54162	2992.625793	2995.51707
-	2993.17884	2991.385776	2988.641087
-	2993.94965	2993.972503	2991.984435
-	2995.32008	2995.105865	2996.330039
-	2997.36905	2996.3502	2993.105352
-	2998.86322	2995.377679	3002.25732
-	3000.10228	3002.461366	2997.257523
-	3001.472	3002.618851	3000.113686
-	3002.96998	3006.309821	2997.178036
-	3004.55833	3008.820217	3008.144022
-	3005.31435	3004.25317	2999.058168
-	3005.95021	3002.335936	2999.04102
-	3007.31039	3007.282447	3005.002555
-	3008.60548	3006.253634	3015.796879
-	3009.42373	3011.659115	3009.859272
-	3010.07295	3010.050657	3012.664186
-	3011.00659	3012.793412	3006.639049
-	3013.51738	3014.788735	3012.559839
-	3012.96949	3010.770864	3016.582434
-	3010.48064	3012.934766	3006.503291
-	3009.4111	3013.644136	3007.680591
-	3009.91011	3008.313801	3011.465451
-	3009.90033	3012.821609	3013.344627
-	3008.68321	3009.001199	3015.467119
-	3012.64739	3013.595362	3011.235266
-	3021.55256	3020.547387	3015.275831
-	3025.4786	3021.998798	3028.268727
-	3029.10614	3026.431998	3025.322537
-	3026.29614	3028.022009	3027.150719
-	3023.39983	3025.529675	3024.178104
-	3026.94464	3028.644062	3023.144129
-	3029.71935	3029.031855	3030.115732
-	3029.53577	3032.877231	3032.152254
-	3029.58918	3033.756322	3031.081613
-	3032.36771	3031.256899	3029.128707
-	3031.22234	3033.931116	3030.048866
-	3034.03544	3036.892891	3033.027063
-	3036.62157	3035.155514	3026.02729
-	3038.57803	3036.201003	3033.003003
-	3037.48512	3038.208166	3034.972299
-	3038.23376	3039.742908	3037.009014
-	3039.75391	3041.273562	3037.948091
-	3042.25516	3040.808764	3038.92502
-	3041.01181	3041.667728	3040.90496
-	3044.8575	3043.903959	3042.940796
-	3048.19083	3046.872149	3047.882974
-	3051.68462	3050.851914	3052.882597
-	3053.34187	3054.063906	3051.863614
-	3055.32908	3052.293279	3053.84869
-	3049.15309	3049.331386	3050.872004
-	3050.35853	3051.11494	3052.780489
-	3053.60842	3054.037481	3055.83
-	3050.36521	3052.912074	3048.654036
-	3042.05465	3045.646515	3042.635671
-	3044.52658	3046.375014	3048.653781
-	3052.32167	3051.437515	3048.597837
-	3066.71786	3069.500018	3063.716725
-	3039.63757	3040.56252	3037.529406
-	3033.44417	3035.625022	3031.574677
-	3028.25761	3028.687524	3027.409126
-	3018.30149	3019.750026	3022.583493
-	3017.96317	3018.812529	3015.795519
-	3028.33176	3027.875031	3025.722446
-	3032.64976	3036.937533	3030.503741
-	3011.1475	3012.000001	3016.424515
-	3009.82976	3005.062503	3010.510866
-	2989.70103	2991.125006	2992.539827
-	2950.21963	2957.187507	2958.464483
-	2860.55231	2851.25001	2869.272897
-	2682.49917	2680.312512	2698.242717
-	2280.78747	2275.375018	2291.227719
-	1544.047	1553.437517	1535.220813
-	756.65396	750.5000194	767.2340739
477.47812	313.03668	312.5625204	320.3064988

**Table A5:** Experimental data for Figure 15.

CO_2_ Injection Time (min)	Viscosity (mPa·s)		
	Experiment #1	Experiment #2	Experiment #3
0	1.2	1.2	1.2
2	269	276	283
4	3123	3052	3079
6	8813	8731	8854
8	10,652	10,511	10,359
10	10,637	10,495	10,372
12	10,612	10,553	10,298
14	10,726	10,527	10,314
16	10,711	10,472	10,381
18	10,694	10,534	10,365
20	10,703	10,526	10,321

**Table A6:** Experimental data for Figure 17.

Salinity (mg·L^−1^)	Viscosity (mPa·s)		
	Experiment #1	Experiment #2	Experiment #3
0	10,310	10,426	10,357
5275.1	10,540	10,615	10,569
21,000	11,300	11,431	11,373
52,780.1	12,090	12,159	12,059
